# Balancing nutrition management and the role of dietitians in eating disorder treatment

**DOI:** 10.1186/s40337-020-00344-x

**Published:** 2020-11-17

**Authors:** Shane Jeffrey, Gabriella Heruc

**Affiliations:** 1 Executive Committee, Australia & New Zealand Academy for Eating Disorders, Sydney, Australia; 2 River Oak Health, Brisbane, Australia; 3 Royal Brisbane and Women’s Hospital, Brisbane, Australia; 4 School of Medicine, Western Sydney University, Campbelltown, Australia; 5 Eating Disorder Service, Northern Sydney Local Health District, Sydney, Australia

**Keywords:** Dietetic, Dietitian, Eating disorder, Malnutrition, Nutrition, Treatment

## Abstract

The symptoms of starvation and dietary restriction are often the subject of targeted intervention in evidence-based treatments across eating disorder diagnoses and treatment models. Despite the level of attention given to these symptoms of clinical malnutrition, they are often treated by health professionals with no nutritional qualifications and in a non-clinical manner in the outpatient setting, with dietitians having no defined role in manualised treatment models. Recently the Australia & New Zealand Academy for Eating Disorders (ANZAED) published practice and training standards for dietitians to help characterise their role in eating disorder treatment. Since malnutrition, secondary to dietary restriction, is a clinically significant nutritional diagnosis that co-occurs in eating disorder presentations, this commentary proposes that dietitians are ideally-positioned to assess and advise on the clinical aspects of malnutrition as a key member of the multidisciplinary team. Food is a central focus in eating disorder treatment, suggesting that nutritional care needs to be addressed by a dietitian alongside the psychological aspects of care that are addressed by a mental health professional.

## Main text

While it is widely accepted that improved nutrition and eating behaviour are an integral aspect of eating disorder treatment, the role of the dietitian in treatment remains unclear. In support of the dietitian’s role, clinical practice guidelines [1, 2] and the clinical practice standards recently published in the Journal of Eating Disorders [3, 4] recommend dietetic involvement in the multidisciplinary eating disorder treatment team. Furthermore, a recent initiative of the Australian Government provided funding under the Medicare Benefits Scheme for eligible patients with an eating disorder diagnosis to access up to twenty sessions with a dietitian per year. However, the manualised, evidence-based treatment models of Family Based Therapy (FBT) [5] and Cognitive Behaviour Therapy – enhanced (CBT-e) [6] make no reference to the direct inclusion of a dietitian in treatment. A recent review of the nutritional content of treatment manuals for adults with an eating disorder found that while 91% of manuals contained some degree of nutritionally-focused content, only 36% of the manuals recommended a dietitian be consulted as part of the multidisciplinary treatment approach [7], suggesting that dietetic involvement is not being recommended for many patients undergoing evidence-based psychological treatment. This is evidenced anecdotally by our own clinical experience, and further supported by a recent delphi study finding that less than 50% of eating disorder specialists agree that patients with an eating disorder should be referred to a dietitian for assessment, education and guidance about nutrition [8]. Since strict *dieting* is noted as a core feature in Fairburn’s transdiagnostic model of eating disorders [9], why is the *dietitian* not integrated into the core of all treatment manuals?

Dietary restriction often demands urgent weight restoration, education about the effects of starvation and minimisation of medical risk. For this reason, it is proposed that when we are treating people with an eating disorder, it is very likely we are also treating clinically significant malnutrition. However, since many eating disorder specialists do not believe dietetic care is necessary for all patients with an eating disorder [8], clinical malnutrition may be inadequately treated *without* referral to a clinician skilled in nutritional management. Furthermore, the clinical skills of the dietitian are superseded in evidence-based treatment manuals by parents who are charged with nutritionally rehabilitating adolescents in FBT and by the mental health professional collaborating with the patient to improve dietary intake in CBT-e. While parents and mental health professionals are well-positioned to help patients eat more, they are not necessarily equipped with the nutritional knowledge about how to treat malnutrition.

Further highlighting the lack of significance placed on the clinical treatment of malnutrition during treatment, McMaster found that of 22 eating disorder treatment manuals, 60% contained nutritional information not substantiated by evidence [7]. Dietitians are highly educated at university in human physiology, biochemistry, pathology and eating behaviour, contributing valuable knowledge and insight to the outpatient treatment team. As such, the dietitian is often viewed as essential in the treatment of malnutrition in other medical conditions [10], as well as in the more intensive eating disorder inpatient and day program treatment settings [11, 12]. However, in outpatient eating disorder treatment, the dietitian’s role becomes less clear and is often missing in evidence-based, manualised treatments. It could be argued in the treatment of anorexia nervosa that weight gain is often used as a proxy measure for nutritional rehabilitation. However, weight gain is a secondary product of nutritional rehabilitation, in which the body is provided with the nutrients to repair, rebuild and improve physiological functioning. Weight gain itself is a very narrow marker of improved health. In treating malnutrition, dietitians go beyond creating an energy surplus to support weight restoration, by also addressing energy availability, the timing and distribution of macronutrients across the day and optimising opportunities to meet micronutrient needs through dietary change [13]. It is this focus on dietary quality, and the role it plays in optimising the nutritional rehabilitation process where dietitians can value add in the course of treatment – elevating malnutrition to the significance it deserves and treating it in a clinically relevant manner. With the goals of supporting nutritional rehabilitation and establishing a positive relationship with food, the nutritional care process has much to offer beyond a focus on weight and eating behaviour. Basing malnutrition solely on current body weight threatens to miss severe life-threatening complications of those with a restrictive eating disorder who are not underweight [14]. Malnutrition is also a potential co-morbid presentation requiring comprehensive assessment for all eating disorder presentations irrespective of diagnosis and body size.

As a member of the multidisciplinary team, the role of the dietitian is to identify the severity of malnutrition, the presence of disordered eating habits, and deficits in nutritional skills and knowledge that inhibit the attainment of adequate nutrition. As outlined in the recently published Australia & New Zealand Academy for Eating Disorders (ANZAED) dietetic practice standards [4], dietitians manage the nutritional care process by facilitating a comprehensive nutritional assessment, formulating a nutritional diagnosis, implementing a nutritional intervention and monitoring progress toward treatment goals through an ongoing evaluation process. Dietitians also assess other common comorbid conditions, such as refeeding syndrome, diabetes mellitus, food allergies, food intolerances, gastrointestinal conditions and osteoporosis. This information is collated to inform the nutritional diagnosis, identifying if the nutritional presentation is associated with eating disorder behaviours and differentiating between disordered eating and that of limited food acceptance.

## Conclusions

In our clinical experience and supported by recent research, malnutrition in outpatient eating disorder presentations is often not clinically assessed or treated in current evidence-based treatment manuals [7], and this is of concern. The idea that dietary restriction is a core behaviour across the eating disorder diagnoses necessitates that nutritional rehabilitation rather than weight restoration alone becomes integrated into outpatient eating disorder treatment approaches. Carolyn Costin stated that, “eating disorders are not about the food, but they are about the food” [15], capturing the need for a multidisciplinary eating disorder treatment team consisting of medical practitioners, mental health professionals *and* dietitians. One way of conceptualising this model is that the medical care of the patient wraps around the psychological and nutritional care processes to ensure the patient is safe to participate in outpatient care. The nutritional and psychological elements of care can be thought of as being connected by a rubber band, moving in tandem and relative to each other during treatment. If the nutritional progress outpaces the psychological progress (as is often the case in inpatient treatment), the rubber band is at risk of breaking, and nutritional progress may not be sustained. Likewise, if psychological progress outpaces nutritional progress, psychological progress may not be sustained due to the persistent effects of starvation [16]. For this reason, both the nutritional and psychological aspects of care warrant the concurrent attention and expertise of the relevant health professionals. The clinical knowledge and skills of nutrition education and counselling are unique to dietitians, making them the ideal professionals to address the nutritional aspects of care to complement psychological evidence-based treatment.

## Data Availability

Not applicable.

## Abbreviations

CBT-e: Cognitive-Behavioral Therapy – enhanced
FBT: Family Based Therapy
